# Supplementary material for: ChatGPT as a Tool for Biostatisticians: A Tutorial on Applications, Opportunities, and Limitations
Source: Stat Med. 2025 Oct 23;44(23-24):e70263. doi: 10.1002/sim.70263 (PMC12548020; doi:10.1002/sim.70263)
Supplement: Supplementary file 1 — Data S1. Supporting Information. [file SIM-44-0-s001.zip › Supplementary Materials Additional.zip!/3.2_Diagnostic_Test/3.2_Diagnostic_Chat8.pdf]

ChatGPT - Diagnostic Testing

I would like to determine sensitivity and specificity of two diagnostic tests. I do have the diagnostic test outcomes, but I do not know the gold standard. What can I do?

Please, tell me more about the latent class analysis.

I have two populations with two different disease prevalences. I have two diagnostic tests which are conditional independent. I would like to get the log-likelihood of this setting.

What is the Hui-Walter paradigm?

I would like to see a worked example and a R script that implements this model.

I would like to add confidence intervals to the estimated sensitivities, specificities, and prevalences.

I would like to run a bootstrap version for robust confidence intervals.

I would like to reparametrize my model using a logistic parametrization for the prevalences, sensitivities, and specificities.

Please update the bootstrap function too.

Could you also perform the calculation of the 95% Confidence intervals via the hessian?

Please provide a comprehensive R code for the issues discussed in this session.

That's an excellent and very well-posed request — you're essentially asking to implement **latent class analysis (LCA)** for **two diagnostic tests**, on **two populations** with differing prevalence, using the **Hui-Walter paradigm**, with:

✅ full likelihood model
✅ parameter estimation (prevalence, sensitivity, specificity)
✅ confidence intervals: profile (via Hessian), and bootstrap
✅ logistic reparametrization for better numerical behavior
✅ full example with R code

🌟 1. Conceptual Background

❓ The Problem

You have two diagnostic tests but **no gold standard**.
You have **two populations** with known different prevalences.

You assume the **two tests are conditionally independent given disease status**.

Goal:
Estimate **sensitivities, specificities**, and **prevalences**, and compute **confidence intervals**.

🧮 Latent Class Analysis (LCA)

LCA allows us to model an **unobserved "latent" variable** (here: true disease status) and explain the **joint distribution of observed test outcomes** as a function of the latent status + test properties.

🧑‍🏫 Hui-Walter Paradigm

Hui & Walter (1980) showed that if:

- You have ≥ 2 populations with different prevalences
- You have ≥ 2 conditionally independent tests
- You model the joint outcomes as a function of sensitivity, specificity, and prevalence

→ then **the model is identifiable**
→ you can **estimate all parameters** by **maximizing the likelihood**.

🧩 Model Setup

---

2 populations: $p = 1, 2$

2 tests: t_1, t_2

Latent variable:

$D \in \{0, 1\}$ true disease status

Observed data:

Each subject has two test results: $(T_1, T_2) \in \{0, 1\}^2$

Parameters:

Parameter	Description	For population p
π_p	Prevalence in population p	Yes
Se_1	Sensitivity of test 1	Global
Sp_1	Specificity of test 1	Global
Se_2	Sensitivity of test 2	Global

Parameter	Description	For population p
Sp_2	Specificity of test 2	Global

Joint probability of outcomes

Assuming **conditional independence**, the probability of observing test pattern (T_1, T_2) in population p is:

$$P(T_1, T_2 \mid p) = \pi_p \cdot P(T_1, T_2 \mid D = 1) + (1 - \pi_p) \cdot P(T_1, T_2 \mid D = 0)$$

with:

$$P(T_1, T_2 \mid D = 1) = Se_1^{T_1}(1 - Se_1)^{1-T_1} \cdot Se_2^{T_2}(1 - Se_2)^{1-T_2}$$
$$P(T_1, T_2 \mid D = 0) = (1 - Sp_1)^{T_1} Sp_1^{1-T_1} \cdot (1 - Sp_2)^{T_2} Sp_2^{1-T_2}$$

📏 Likelihood

You observe counts n_{p,T_1,T_2} in each population for each pattern.

Then the **log-likelihood** is:

$$\log L = \sum_{p=1}^{2} \sum_{T_1=0}^{1} \sum_{T_2=0}^{1} n_{p,T_1,T_2} \cdot \log P(T_1, T_2 \mid p)$$

🚀 Logistic Reparametrization

To ensure parameters stay in valid ranges (0,1), we model:

$$\pi_p = \frac{1}{1 + \exp(-\theta_{\pi_p})}$$
$$Se_j = \frac{1}{1 + \exp(-\theta_{Se_j})}$$
$$Sp_j = \frac{1}{1 + \exp(-\theta_{Sp_j})}$$

🧑‍💻 Full R Implementation

Now I will provide a full worked example:

📊 1️⃣ Simulated Example

```r
# Load libraries
library(stats)
```

```
library(numDeriv)  # for Hessian
library(boot)      # for bootstrap

# Simulate data
set.seed(123)

# True parameters
true_params <- list(
  pi1 = 0.3,
  pi2 = 0.6,
  Se1 = 0.85,
  Sp1 = 0.90,
  Se2 = 0.80,
  Sp2 = 0.95
)

# Function to simulate data for one population
simulate_population <- function(N, pi, Se1, Sp1, Se2, Sp2) {
  D <- rbinom(N, 1, pi)
  T1 <- ifelse(D == 1, rbinom(N, 1, Se1), rbinom(N, 1, 1 - Sp1))
  T2 <- ifelse(D == 1, rbinom(N, 1, Se2), rbinom(N, 1, 1 - Sp2))
  table(factor(T1, levels=0:1), factor(T2, levels=0:1))
}

# Sample sizes
N1 <- 500
N2 <- 500

# Simulate data for both populations
table1 <- simulate_population(N1, true_params$pi1, true_params$Se1, true_params$Sp1,
true_params$Se2, true_params$Sp2)
table2 <- simulate_population(N2, true_params$pi2, true_params$Se1, true_params$Sp1,
true_params$Se2, true_params$Sp2)

# Convert to vector of counts: (00, 01, 10, 11)
counts1 <- as.vector(table1)
counts2 <- as.vector(table2)

# Combine data
counts <- c(counts1, counts2)
```

🧮 2️⃣ Log-Likelihood Function with Logistic Reparametrization

```r
# Log-likelihood function
loglik_fun <- function(theta, counts) {
  # Unpack parameters
  pi1 <- 1 / (1 + exp(-theta[1]))
  pi2 <- 1 / (1 + exp(-theta[2]))
  Se1 <- 1 / (1 + exp(-theta[3]))
  Sp1 <- 1 / (1 + exp(-theta[4]))
  Se2 <- 1 / (1 + exp(-theta[5]))
  Sp2 <- 1 / (1 + exp(-theta[6]))

  # Probabilities of patterns (00,01,10,11) in population 1
  P1 <- numeric(4)
  P1[1] <- pi1 * (1-Se1)*(1-Se2) + (1-pi1) * Sp1 * Sp2
  P1[2] <- pi1 * (1-Se1)*Se2     + (1-pi1) * Sp1 * (1-Sp2)
  P1[3] <- pi1 * Se1 * (1-Se2)   + (1-pi1) * (1-Sp1) * Sp2
  P1[4] <- pi1 * Se1 * Se2       + (1-pi1) * (1-Sp1) * (1-Sp2)

  # Same for population 2
  P2 <- numeric(4)
  P2[1] <- pi2 * (1-Se1)*(1-Se2) + (1-pi2) * Sp1 * Sp2
  P2[2] <- pi2 * (1-Se1)*Se2     + (1-pi2) * Sp1 * (1-Sp2)
  P2[3] <- pi2 * Se1 * (1-Se2)   + (1-pi2) * (1-Sp1) * Sp2
  P2[4] <- pi2 * Se1 * Se2       + (1-pi2) * (1-Sp1) * (1-Sp2)

  # Log-likelihood
```

```
  logL <- sum(counts[1:4] * log(P1)) + sum(counts[5:8] * log(P2))
  return(-logL)  # negative log-likelihood for minimization
}
```

🏃 3️⃣ Optimization

```r

# Initial values on logit scale (0 = logit(0.5))
init_theta <- rep(0, 6)

# Optimize
fit <- optim(init_theta, loglik_fun, counts=counts, method="BFGS", hessian=TRUE)

# Estimated parameters
theta_hat <- fit$par

# Back-transform
pi1_hat <- 1 / (1 + exp(-theta_hat[1]))
pi2_hat <- 1 / (1 + exp(-theta_hat[2]))
Se1_hat <- 1 / (1 + exp(-theta_hat[3]))
Sp1_hat <- 1 / (1 + exp(-theta_hat[4]))
Se2_hat <- 1 / (1 + exp(-theta_hat[5]))
Sp2_hat <- 1 / (1 + exp(-theta_hat[6]))

# Print estimates
estimates <- c(pi1=pi1_hat, pi2=pi2_hat, Se1=Se1_hat, Sp1=Sp1_hat, Se2=Se2_hat, Sp2=Sp2_hat)
print(estimates)
```

📏 4️⃣ Confidence Intervals via Hessian

```r

# Variance-covariance matrix
vcov <- solve(fit$hessian)

# Standard errors (on logit scale)
se_theta <- sqrt(diag(vcov))

# Delta method: transform to probability scale
logit_to_prob <- function(theta, se_theta) {
  p <- 1 / (1 + exp(-theta))
  dp_dtheta <- p * (1 - p)
  se_p <- dp_dtheta * se_theta
  ci_lower <- p - 1.96 * se_p
  ci_upper <- p + 1.96 * se_p
  return(c(estimate=p, lower=ci_lower, upper=ci_upper))
}

# Apply to each parameter
cis <- t(sapply(1:6, function(i) logit_to_prob(theta_hat[i], se_theta[i])))
rownames(cis) <- c("pi1", "pi2", "Se1", "Sp1", "Se2", "Sp2")
print(cis)
```

🔄 5️⃣ Bootstrap Confidence Intervals

```r

# Bootstrap function
bootstrap_fun <- function(counts, indices) {
  # Resample counts with replacement within each population
```

```r
  boot_counts <- counts
  for (pop in 1:2) {
    idx <- (1:4) + (pop-1)*4
    boot_sample <- rmultinom(1, sum(counts[idx]), counts[idx] / sum(counts[idx]))
    boot_counts[idx] <- boot_sample
  }

  # Refit model
  fit_boot <- optim(init_theta, loglik_fun, counts=boot_counts, method="BFGS")
  theta_b <- fit_boot$par

  # Return back-transformed estimates
  return(c(
    1 / (1 + exp(-theta_b[1])),  # pi1
    1 / (1 + exp(-theta_b[2])),  # pi2
    1 / (1 + exp(-theta_b[3])),  # Se1
    1 / (1 + exp(-theta_b[4])),  # Sp1
    1 / (1 + exp(-theta_b[5])),  # Se2
    1 / (1 + exp(-theta_b[6]))   # Sp2
  ))
}

# Run bootstrap
n_boot <- 1000
boot_results <- replicate(n_boot, bootstrap_fun(counts, NULL))

# Compute percentile CIs
boot_cis <- apply(boot_results, 1, function(x) quantile(x, c(0.025, 0.975)))
rownames(boot_cis) <- c("2.5%", "97.5%")
colnames(boot_cis) <- c("pi1", "pi2", "Se1", "Sp1", "Se2", "Sp2")
print(boot_cis)
```

🚀 Summary

✅ **Full likelihood using Hui-Walter paradigm**
✅ **Logistic parametrization**
✅ **MLE with BFGS**
✅ **Confidence intervals from Hessian**
✅ **Bootstrap for robust CIs**

If you want, I can also provide:

1️⃣ **Bayesian version (with `rstan` or `JAGS`)**
2️⃣ **Extension to > 2 populations, > 2 tests**
3️⃣ **Allowing conditional dependence (covariance terms)**

Would you like me to generate a **single complete R script** with everything combined in a clear, reusable way? Just say: **"Yes, full script please"** 🚀.

Please ürpvode a single complete R code

Here is a **single, complete, well-structured R script** that implements the **entire Hui-Walter paradigm with two tests, two populations, logistic parametrization, CIs from Hessian, and bootstrap**:

🌟 FULL R SCRIPT: Hui-Walter Paradigm with LCA

```r
# ==========================================
# Hui-Walter Latent Class Analysis - FULL EXAMPLE
# Two tests, two populations
# Logistic parametrization
# Confidence intervals via Hessian and Bootstrap
# ==========================================

# Load required libraries
library(numDeriv)  # for Hessian
library(boot)      # for bootstrap

# ==========================================
# 1️⃣ Simulate Data
# ==========================================

# True parameters
true_params <- list(
  pi1 = 0.3,
  pi2 = 0.6,
  Se1 = 0.85,
  Sp1 = 0.90,
  Se2 = 0.80,
  Sp2 = 0.95
)

# Function to simulate one population
simulate_population <- function(N, pi, Se1, Sp1, Se2, Sp2) {
  D <- rbinom(N, 1, pi)
  T1 <- ifelse(D == 1, rbinom(N, 1, Se1), rbinom(N, 1, 1 - Sp1))
  T2 <- ifelse(D == 1, rbinom(N, 1, Se2), rbinom(N, 1, 1 - Sp2))
  table(factor(T1, levels=0:1), factor(T2, levels=0:1))
}

# Sample sizes
N1 <- 500
N2 <- 500

# Simulate both populations
set.seed(123)
table1 <- simulate_population(N1, true_params$pi1, true_params$Se1, true_params$Sp1,
true_params$Se2, true_params$Sp2)
table2 <- simulate_population(N2, true_params$pi2, true_params$Se1, true_params$Sp1,
true_params$Se2, true_params$Sp2)

# Convert tables to vector: (00, 01, 10, 11)
counts1 <- as.vector(table1)
counts2 <- as.vector(table2)
counts <- c(counts1, counts2)

cat("Observed counts per pattern:\n")
print(matrix(counts, nrow=4, byrow=FALSE, dimnames=list(c("00","01","10","11"),
c("Pop1","Pop2"))))

# ==========================================
# 2️⃣ Log-Likelihood Function
# ==========================================

loglik_fun <- function(theta, counts) {
  # Unpack parameters
  pi1 <- 1 / (1 + exp(-theta[1]))
  pi2 <- 1 / (1 + exp(-theta[2]))
  Se1 <- 1 / (1 + exp(-theta[3]))
  Sp1 <- 1 / (1 + exp(-theta[4]))
  Se2 <- 1 / (1 + exp(-theta[5]))
  Sp2 <- 1 / (1 + exp(-theta[6]))

  # Probabilities for Pop1
  P1 <- numeric(4)
  P1[1] <- pi1 * (1-Se1)*(1-Se2) + (1-pi1) * Sp1 * Sp2
```

```
  P1[2] <- pi1 * (1-Se1)*Se2     + (1-pi1) * Sp1 * (1-Sp2)
  P1[3] <- pi1 * Se1 * (1-Se2)   + (1-pi1) * (1-Sp1) * Sp2
  P1[4] <- pi1 * Se1 * Se2       + (1-pi1) * (1-Sp1) * (1-Sp2)

  # Probabilities for Pop2
  P2 <- numeric(4)
  P2[1] <- pi2 * (1-Se1)*(1-Se2) + (1-pi2) * Sp1 * Sp2
  P2[2] <- pi2 * (1-Se1)*Se2     + (1-pi2) * Sp1 * (1-Sp2)
  P2[3] <- pi2 * Se1 * (1-Se2)   + (1-pi2) * (1-Sp1) * Sp2
  P2[4] <- pi2 * Se1 * Se2       + (1-pi2) * (1-Sp1) * (1-Sp2)

  # Total log-likelihood
  logL <- sum(counts[1:4] * log(P1)) + sum(counts[5:8] * log(P2))

  return(-logL)  # Negative for minimization
}

# ==========================================
# 3️⃣ Parameter Estimation (MLE)
# ==========================================

# Initial values (logit scale)
init_theta <- rep(0, 6)

# Run optimizer
fit <- optim(init_theta, loglik_fun, counts=counts, method="BFGS", hessian=TRUE,
control=list(maxit=10000))

# Estimated parameters
theta_hat <- fit$par

# Back-transform to probability scale
pi1_hat <- 1 / (1 + exp(-theta_hat[1]))
pi2_hat <- 1 / (1 + exp(-theta_hat[2]))
Se1_hat <- 1 / (1 + exp(-theta_hat[3]))
Sp1_hat <- 1 / (1 + exp(-theta_hat[4]))
Se2_hat <- 1 / (1 + exp(-theta_hat[5]))
Sp2_hat <- 1 / (1 + exp(-theta_hat[6]))

estimates <- c(pi1=pi1_hat, pi2=pi2_hat, Se1=Se1_hat, Sp1=Sp1_hat, Se2=Se2_hat, Sp2=Sp2_hat)
cat("\nMLE Estimates:\n")
print(estimates)

# ==========================================
# 4️⃣ Confidence Intervals via Hessian
# ==========================================

# Variance-covariance matrix
vcov <- solve(fit$hessian)

# Standard errors on logit scale
se_theta <- sqrt(diag(vcov))

# Delta method: transform to probability scale
logit_to_prob <- function(theta, se_theta) {
  p <- 1 / (1 + exp(-theta))
  dp_dtheta <- p * (1 - p)
  se_p <- dp_dtheta * se_theta
  ci_lower <- p - 1.96 * se_p
  ci_upper <- p + 1.96 * se_p
  return(c(estimate=p, lower=ci_lower, upper=ci_upper))
}

# Apply to all parameters
cis_hessian <- t(sapply(1:6, function(i) logit_to_prob(theta_hat[i], se_theta[i])))
rownames(cis_hessian) <- c("pi1", "pi2", "Se1", "Sp1", "Se2", "Sp2")

cat("\nConfidence intervals via Hessian (Delta method):\n")
print(cis_hessian)

# ==========================================
# 5️⃣ Bootstrap Confidence Intervals
# ==========================================

bootstrap_fun <- function(counts, indices) {
```

```
  # Resample counts within each population
  boot_counts <- counts
  for (pop in 1:2) {
    idx <- (1:4) + (pop-1)*4
    boot_sample <- rmultinom(1, sum(counts[idx]), counts[idx] / sum(counts[idx]))
    boot_counts[idx] <- boot_sample
  }

  # Refit model
  fit_boot <- optim(init_theta, loglik_fun, counts=boot_counts, method="BFGS",
control=list(maxit=10000))
  theta_b <- fit_boot$par

  # Return back-transformed estimates
  return(c(
    1 / (1 + exp(-theta_b[1])),  # pi1
    1 / (1 + exp(-theta_b[2])),  # pi2
    1 / (1 + exp(-theta_b[3])),  # Se1
    1 / (1 + exp(-theta_b[4])),  # Sp1
    1 / (1 + exp(-theta_b[5])),  # Se2
    1 / (1 + exp(-theta_b[6]))   # Sp2
  ))
}

# Run bootstrap
n_boot <- 1000
cat("\nRunning bootstrap... (may take a moment)\n")
set.seed(456)
boot_results <- replicate(n_boot, bootstrap_fun(counts, NULL))

# Compute percentile bootstrap CIs
boot_cis <- apply(boot_results, 1, function(x) quantile(x, c(0.025, 0.975)))
rownames(boot_cis) <- c("2.5%", "97.5%")
colnames(boot_cis) <- c("pi1", "pi2", "Se1", "Sp1", "Se2", "Sp2")

cat("\nBootstrap percentile confidence intervals:\n")
print(boot_cis)

# ==========================================
# DONE 🚀
# ==========================================
```

✅ What this code does:

✅ Simulates 2 populations under true parameters
✅ Builds **log-likelihood** with logistic parametrization
✅ Runs **MLE** with **`optim`** + **Hessian-based CIs**
✅ Runs **bootstrap** and computes **bootstrap CIs**
✅ Clean output and flexible structure

🚀 How to run:

Just copy the full script into a file like:

```
bash

hui_walter_lca.R
```

And run in R / RStudio:

```r
source("hui_walter_lca.R")
```

📢 Notes:

- It is easily extendable to **more tests**, **more populations**.
- You can add **covariance terms** for **conditional dependence** if needed.
- I can also generate **Bayesian versions** (with Stan or JAGS) if you'd like.

Would you like me to generate next:

1️⃣ **Bayesian version**
2️⃣ **Model allowing conditional dependence**
3️⃣ **Generalization to more than 2 tests**

Just tell me: **"Next step: ..."** 🚀.
